# Epigallocatechin gallate incorporation into lignin enhances the alkaline delignification and enzymatic saccharification of cell walls

**DOI:** 10.1186/1754-6834-5-59

**Published:** 2012-08-13

**Authors:** Sasikumar Elumalai, Yuki Tobimatsu, John H Grabber, Xuejun Pan, John Ralph

**Affiliations:** 1 Department of Biological Systems Engineering, University of Wisconsin-Madison, 460 Henry Mall, Madison, WI, 53706, USA; 2 Department of Biochemistry, University of Wisconsin-Madison, 433 Babcock Drive, Madison, WI, 53706, USA; 3 U.S. Dairy Forage Research Center, USDA-Agricultural Research Service, 1925 Linden Drive West, Madison, WI, 53706, USA; 4 DOE Great Lakes Bioenergy Research Center, and Wisconsin Bioenergy Initiative, University of Wisconsin-Madison, Madison, WI, 53706, USA

**Keywords:** Lignification, Radical cross-coupling, Digestibility, Pretreatment, Saccharification

## Abstract

**Background:**

Lignin is an integral component of the plant cell wall matrix but impedes the conversion of biomass into biofuels. The plasticity of lignin biosynthesis should permit the inclusion of new compatible phenolic monomers such as flavonoids into cell wall lignins that are consequently less recalcitrant to biomass processing. In the present study, epigallocatechin gallate (EGCG) was evaluated as a potential lignin bioengineering target for rendering biomass more amenable to processing for biofuel production.

**Results:**

*In vitro* peroxidase-catalyzed polymerization experiments revealed that both gallate and pyrogallyl (B-ring) moieties in EGCG underwent radical cross-coupling with monolignols mainly by β–O–4-type cross-coupling, producing benzodioxane units following rearomatization reactions. Biomimetic lignification of maize cell walls with a 3:1 molar ratio of monolignols and EGCG permitted extensive alkaline delignification of cell walls (72 to 92%) that far exceeded that for lignified controls (44 to 62%). Alkali-insoluble residues from EGCG-lignified walls yielded up to 34% more glucose and total sugars following enzymatic saccharification than lignified controls.

**Conclusions:**

It was found that EGCG readily copolymerized with monolignols to become integrally cross-coupled into cell wall lignins, where it greatly enhanced alkaline delignification and subsequent enzymatic saccharification. Improved delignification may be attributed to internal trapping of quinone-methide intermediates to prevent benzyl ether cross-linking of lignin to structural polysaccharides during lignification, and to the cleavage of ester intra-unit linkages within EGCG during pretreatment. Overall, our results suggest that apoplastic deposition of EGCG for incorporation into lignin would be a promising plant genetic engineering target for improving the delignification and saccharification of biomass crops.

## Background

**Figure 1:**
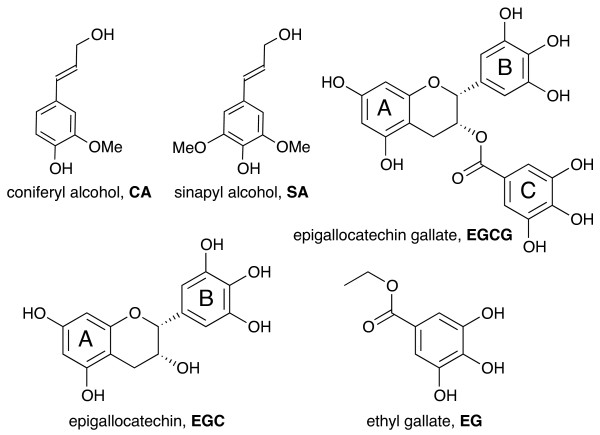
Structures of conventional monolignols, coniferyl alcohol (CA) and sinapyl alcohol (SA), and flavonols and gallate derivatives used in this study, epigallocatechin gallate (EGCG), epigallocatechin (EGC), and ethyl gallate (EG).

Lignin serves a vital role as an inter- and intra-molecular glue strengthening plant cell walls, but it hinders numerous agro-industrial processes such as the chemical pulping of woody crops, forage digestion by livestock, and the enzymatic saccharification and fermentation of lignocellulosic biomass into liquid biofuels. As a result, considerable effort has been directed towards reducing or altering the biosynthesis of lignin in plants to permit more efficient utilization of plant cell walls [1-7]. In angiosperms, lignin is normally formed by the oxidative copolymerization of monolignols, principally coniferyl alcohol (CA) and sinapyl alcohol (SA), Figure 1. Perturbing single or multiple genes in the monolignol pathway can lead to massive structural changes in the polymer due to dramatic shifts in the deposition of normal monolignols [8-11], and/or incorporation of pathway intermediates and other phenolic compounds [12-16]. The inherent malleability of plant lignification is further illustrated by the natural incorporation of various γ-acylated monolignols [13,17,18] and ferulate arabinoxylan esters [19,20] into lignin and the recent discovery of a seed-coat lignin surprisingly formed solely from caffeyl alcohol [21]. These findings support the notion that plants could be genetically engineered to make use of precursors from alternate phenolic pathways to form lignins that are more amenable to processing [20,22-30].

In order to identify the most promising genetic engineering targets for modifying lignin, we have used a biomimetic cell wall model system to test a variety of plant-derived phenolics as alternative precursors for lignification [31-34]. These studies have demonstrated that copolymerization of hydroxycinnamate conjugates such as coniferyl ferulate [34] and rosmarinic acid [31] with normal monolignols dramatically improves the alkali extractability of lignin and the subsequent enzymatic hydrolysis of fiber. Accompanying *in vitro* lignification studies demonstrated that these conjugates readily participate in peroxidase-catalyzed copolymerization reactions with normal monolignols. The resulting lignin contains readily cleaved ester linkages in the backbone of the polymer which permit lignin depolymerization under mild alkaline conditions [31]. Subsequent cell wall studies revealed that several flavonoid and gallate derivatives hold promise as monolignol substitutes for modulating the adverse effects of lignin to enhance the inherent fermentability of cell walls [32,33]. Among these, epigallocatechin gallate (EGCG, Figure 1) was particularly attractive because it readily formed wall-bound polymers with normal monolignols and enhanced the fermentability of non-pretreated cell walls by 25% [32]. Similarly to the aforementioned hydroxycinnamate conjugates, incorporation of EGCG could introduce easily cleaved ester linkages into the lignin backbone via oxidative coupling of its epigallocatechin and gallate moieties with monolignols. However, the involvement of these EGCG moieties in coupling reactions with monolignols is not known. It is also not known whether EGCG incorporation into lignin could enhance the delignification of cell walls by chemical pretreatment and/or their saccharification by hydrolytic enzymes.

Therefore in the present study, we examined the copolymerization of EGCG and CA into dehydrogenation polymers (synthetic lignins, DHPs), utilizing an *in vitro* horseradish peroxidase (HRP)-catalyzed polymerization system that models lignin polymerization *in vivo *[35-38]. Two-dimensional nuclear magnetic resonance (NMR) experiments with the DHPs revealed one major cross-coupling mode between CA and both epigallocatechin and gallate moieties of EGCG. We then subjected cell wall dehydrogenation polymers (CWDHPs) formed by artificially lignifying maize cell walls with CA, SA, and EGCG [32] to alkaline pretreatment and enzymatic hydrolysis. Wet chemical and NMR analyses of CWDHPs, alkali-insoluble residues, and enzyme hydrolysates revealed that EGCG incorporation into lignin dramatically enhanced the delignification and enzymatic saccharification of cell walls.

## Results and discussion

### *In vitro* lignin polymerization with EGCG

In these experiments, we examined HRP/H_2_O_2_-mediated coupling reactions of EGCG and simplified models of its gallate and gallocatechin moieties with CA, a conventional plant monolignol (Figure 1). To avoid excess formation of insoluble polymers that are difficult to analyze by NMR, most copolymerization reactions were quickly quenched after 10 min of reaction time. Soluble fractions consisting mainly of low molecular weight polymerization products were then extracted with ethyl acetate or acetone in yields ranging from 42-55% for subsequent NMR analysis (see Materials and Methods). Based on thin layer chromatography, the soluble fractions contained only coupling products and no unreacted monomers.

**Figure 2:**
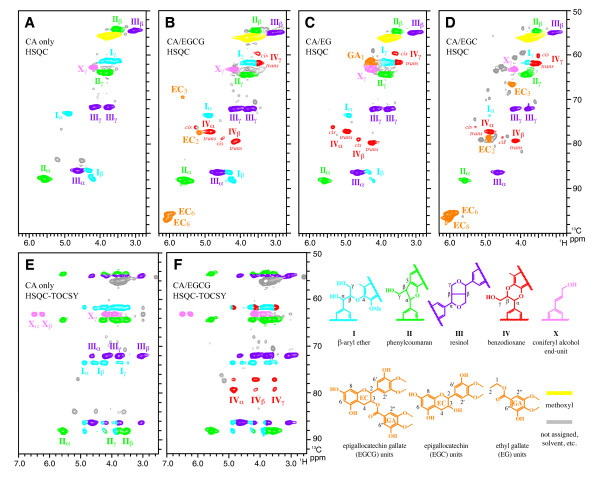
**(A-D) Short-range**^** 13**^**C–**^**1**^**H correlation (HSQC) NMR spectra of* in vitro *peroxidase-catalyzed polymerization products from coniferyl alcohol only (A), and from coniferyl alcohol with epigallocatechin gallate (B), epigallocatechin (C), and ethyl gallate (D). (E** and **F)** Short-range ^13^C–^1^H total correlation (HSQC-TOCSY) spectra of the polymerization products from coniferyl alcohol only **(E)**, and from coniferyl alcohol with epigallocatechin gallate **(F)**.

Detailed chemical structures of the polymerization products were elucidated by 2D NMR methods. The HSQC spectra resolved signatures of the various inter-unit linkage types in the oxidation products and clearly revealed the participation of EGCG, epigallocatechin (EGC), and ethyl gallate (EG) in lignin polymerization with CA (Figures 2A-D). In agreement with literature data [39,40], the polymerization products prepared only with CA contained mainly phenylcoumaran units **II** with moderate levels of β-aryl ether units **I** and resinol units **III** (Figure 2A). Signals from the complete side-chains of these units were seen in the 2D HSQC-TOCSY spectrum (Figure 2E). Such typical lignin units, representing these standard linkage types, were also visible in the spectra of the oxidation products prepared with EGCG, but the most striking difference was the appearance of benzodioxane units **IV** (Figure 2B), which were totally absent in the control (Figure 2A). The α-, β-, and γ-correlations from *trans*-benzodioxane-ring units could be assigned by comparison with literature data of analogous benzodioxanes [21,31] and were further authenticated by TOCSY (Figure 2F). Less pronounced correlations from presumed *cis*-benzodioxane-ring units were also visible. Benzodioxane structures were also evident in oxidations carried out with EG and EGC (Figure 2, C and D), demonstrating that both epigallocatechin and gallate moieties comprising EGCG could participate in cross-coupling reactions with CA. It is likely that the gallate moiety participated in lignin polymerization primarily via the benzodioxane pathway, because EG polymerization products contained only benzodioxane signals besides the typical lignin signals from CA (Figure 2C). On the other hand, the spectrum of EGC polymerization products (Figure 2D) was complex with numerous unidentified signals (colored in grey), suggesting that the epigallocatechin moiety in EGCG might be involved in several cross-coupling modes besides the pathway to benzodioxanes. In both EGCG and EGC spectra, the two signals from unreacted free resorcinol A rings (EC_6_ and EC_8_) were especially prominent, suggesting that reactions of pyrogallol B and gallate rings far exceed reactions of the resorcinol A-ring. Previous studies examining chemical and enzymatic oxidations of flavonols in the absence of monolignols reported lower reactivity of A-ring in comparison to B-ring for EGCG and analogous compounds [41-44].

We also examined synthetic lignins (DHPs) prepared by the conventional *in vitro* lignin polymerization method (end-wise polymerization method), in which the monomers and hydrogen peroxide solutions were slowly added (~20 h) to the peroxidase solution to facilitate polymer chain elongation [45,46]. These experiments produced DHPs from CA and EGCG (10–20 mol%, in the monomer feed) in good yields (70-80%) but, unlike traditional DHPs prepared only with CA, DHPs prepared with EGCG were mostly insoluble in common lignin solvents used for solution-state NMR. Nevertheless, the HSQC spectrum of the DHP acquired in a suspension-state in dimethylsulfoxide-*d*_6_/pyridine-*d*_5_ (4:1, v/v) contained signals diagnostic of EGCG aromatic rings and benzodioxane units (Additional file 1: Figure S1); weak signals are likely due to the poor solubility of EGCG-containing polymers. Overall these studies suggest that EGCG readily undergoes oxidative coupling with normal monolignols to produce polymeric lignin.

**Figure 3:**
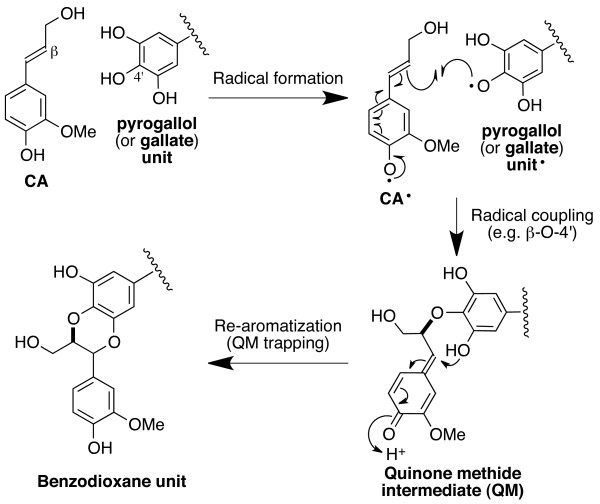
**Generation of benzodioxane units during lignification via β–O–4′-cross-coupling reactions between a monolignol (shown here with coniferyl alcohol only) and pyrogallol or gallate units in epigallocatechin gallate.** Internal trapping of the intermediate quinone methide by the *o*-phenol allows rapid rearomatization without requiring external nucleophilic attack (by water, polysaccharide hydroxyls, or other nucleophiles).

Various flavonol and gallate derivatives including EGCG are known to undergo oxidative homo-coupling reactions in the presence of enzymatic and chemical oxidants [41-44]. When such oxidations are carried out in the presence of monolignols, preferential cross-coupling of galloyl phenoxy radicals from EGCG with monolignol β-radicals and subsequent internal trapping of the quinone methide intermediates (QM) generates benzodioxanes; a logical pathway of such EGCG-incorporation is shown in Figure 3. Cross-coupling between monolignols and galloyl compounds have been implicated but not demonstrated in previous *in vivo* and *in vitro* studies [47,48]. Thus, we are the first to confirm that gallate and pyrogallyl compounds readily participate in lignin polymerization reactions to generate benzodioxane units. Analogous benzodioxanes have also been authenticated as products of lignification with other atypical lignin precursors with *o*-diphenolic structures such as caffeyl alcohol [16,21], 5-hydroxyconiferyl alcohol [14,15,24,49], and rosmarinic acid [31], either *in vivo* or *in vitro*, or both. Overall, the data presented here support our earlier contention that EGCG readily undergoes oxidative coupling reactions with monolignols to produce polymers with readily cleavable ester linkages in the polymer backbone [32,33]. Extensive formation of benzodioxane structures should also block the formation of lignin-carbohydrate cross-links formed via lignin quinone methide intermediates because quinone methide intermediates are readily trapped internally by the *ortho*-OH (Figure 3) and are therefore not available for trapping by external nucleophiles such as water or hydroxyls on polysaccharides [19,50].

### Alkaline delignification of cell walls lignified with EGCG

To examine the impact of EGCG on the formation of lignin in cell walls and its removal by alkaline pretreatments, we prepared cell wall-dehydrogenation polymer (CWDHP) complexes by adding dilute H_2_O_2_ and lignin precursors to non-lignified primary walls of maize containing natively bound peroxidase. When added, EGCG comprised about 45% by weight of the precursor mixture. The resulting CWDHP control prepared with CA and SA and the CWDHP-EGCG complex prepared with CA, SA, and EGCG contained similar amounts of lignin (averaging of 19.3%) as determined by the acetyl bromide method (Table 1). Comparable lignin concentrations were previously obtained by the Klason lignin procedure and by mass balance calculations [32], indicating that EGCG readily formed wall-bound polymers with normal monolignols. Both CWDHPs contained about 30% glucose (derived mainly from cellulose) and high proportions of the non-cellulosic sugars (xylose, arabinose, and galactose) that are characteristic of primary cell walls in grasses [51].

**Table 1:** Composition of cell-wall dehydrogenation polymers (CWDHPs) and their alkali-insoluble residues

**CWDHP**	**Alkaline pretreatment (°C)**	**Concentration in CWDHP or alkali-insoluble residues (%)**				
		**Lignin**	**Glucose**	**Xylose**	**Arabinose**	**Galactose**
Control	None	20.2a^a^	29.6 g	x11.5b	16.2b	7.3bc
	70	16.2b	32.0f	11.4b	16.5b	8.0a
	100	17.6b	34.0e	11.4 b	16.5b	7.1d
	130	16.9b	35.9d	12.9a	18.3a	5.6e
EGCG	None	18.4ab	30.4 fg	11.5b	16.0b	7.4b
	70	8.9c	40.9c	11.6b	14.9 c	7.1 cd
	100	6.5d	43.5b	9.9c	12.3d	7.2 cd
	130	5.5d	48.8a	9.7c	10.8e	4.4f
Analysis of variance						
CWDHP		†	***	***	***	***
Pretreatment		***	***	**	**	***
CWDHP X Pretreatment Interaction		***	***	***	***	***

The CWDHP were formed with monolignols (control) or with monolignols plus epigallocatechin gallate (EGCG) and pretreated with 0.075 M sodium hydroxide (15% w/w loading) at various temperatures.

^a^ Means within columns with unlike letters differ (P < 0.05).

† *P* < 0.1; * *P* < 0.05; ** *P* < 0.01, *** *P* < 0.001.

To assess EGCG effects on cell wall delignification and carbohydrate recovery, CWDHPs were subjected to pretreatment with aqueous NaOH (15% w/w loading on CWDHPs) at 70, 100, and 130°C and alkali-insoluble residues were analyzed by wet-chemical methods and by gel-state 2D NMR. Alkaline pretreatments substantially decreased lignin and non-cellulosic sugar concentrations and increased glucose concentrations in residues recovered from CWDHP-EGCG, whereas comparatively modest compositional shifts were observed following alkaline pretreatment of the CWDHP control (Table 1).

Recovery of alkali-insoluble residues from CWDHP-EGCG was 15 to 20 percentage points lower than from CWDHP-control (Table 2). Based on the recovery of residues and their composition, the incorporation of EGCG increased the extractability of lignin at each pretreatment temperature by an average of 32 percentage points compared to the control (Table 2). Thus ECGC incorporation into lignin permitted more extensive delignification of cell walls under milder conditions that what would be possible for cell walls lignified with normal monolignols. For example, alkaline pretreatment at 130°C removed 62% of the lignin from CWDHP controls, while a less severe alkaline pretreatment at 70°C removed 72% of the lignin from CWDHP-EGCG. Increasing pretreatment temperature to 100 or 130°C boosted delignification of CWDHP-EGCG to about 90%, far in excess of that realized for CWDHP controls.

**Table 2:** Proportion of lignin removed and recovery of alkali-insoluble residues and cell wall sugars from cell wall dehydrogenation polymers (CWDHPs)

**CWDHP**	**Alkaline pretreatment (°C)**	**Lignin extracted (%)**	**Recovered (%)**					
			**Residue**	**Glucose**	**Xylose**	**Arabinose**	**Galactose**	**Total sugars**
Control	70	43.7d	70.4a	76.0a	69.7a	72.2a	77.8a	74.2a
	100	49.7d	57.6b	66.1b	56.7b	59.1b	56.2b	61.6b
	130	61.9c	45.6c	55.2c	51.1c	51.7c	35.2c	51.5c
EGCG	70	72.4b	56.7b	76.4a	57.5b	52.7c	54.5b	64.9b
	100	87.1a	36.3d	52.1c	31.2d	28.0d	35.2c	40.7d
	130	92.4a	25.6e	41.2d	21.6e	17.2e	15.3d	29.0e
Analysis of variance								
CWDHP		*	***	***	***	***	***	***
Pretreatment		***	***	***	***	***	***	***
CWDHP X Pretreatment Interaction		NS	*	**	***	***	NS	**

The CWDHP were formed with monolignols (control) or with monolignols plus epigallocatechin gallate (EGCG) and pretreated with 0.075 M sodium hydroxide (15% w/w loading) at various temperatures.

^a^ Means within columns with unlike letters differ (P < 0.05).

NS not significant; * *P* < 0.05; ** *P* < 0.01, *** *P* < 0.001.

**Figure 4:**
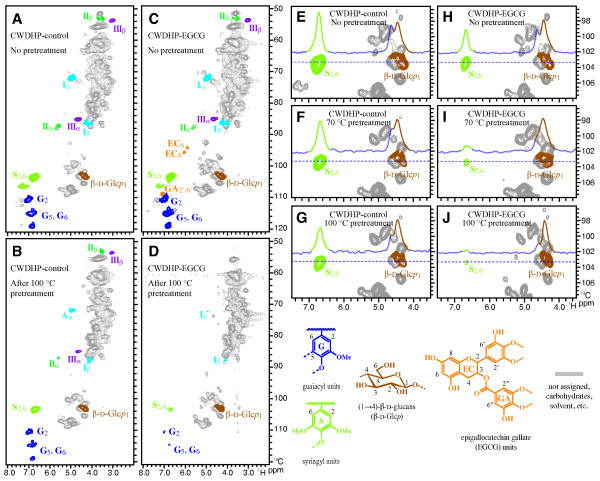
**Gel-state 2D**^** 13**^**C–**^**1**^**H correlation (HSQC) NMR spectra of maize cell walls lignified with coniferyl alcohol and sinapyl alcohol only (CWDHP-control), and in combination with epigallocatechin gallate (CWDHP-EGCG).** (**A**-**D**) Partial HSQC spectra of CWDHP-control (**A** and **B**) and CWDHP-EGCG (**C** and **D**) before and after the alkali pretreatment at 100°C. (**E**-**J**) Expanded HSQC spectra and sliced 1D F2 (^1^H) spectra (at 103.3 ppm in F1, ^13^C) showing syringyl lignin aromatic (S_2/6_) and glucan anomeric (β-D-Glc*p*_1_) signals, of CWDHP-control (**E**-**G**) and CWDHP-EGCG (**H**-**J**) before and after the alkali pretreatment at 70°C, 100°C, and 130°C.

Gel-state NMR of CWDHPs and their alkali-insoluble residues confirmed that copolymerization of monolignols with EGCG dramatically enhanced the alkaline extractability of lignin (Figure 4). Alkaline pretreatment slightly reduced all visible lignin signals in the CWDHP-control spectra (Figure 4 A and B), but substantially reduced lignin signals in CWDHP-EGCG spectra (Figure 4 C and D). Sliced 1D F2 (^1^H) spectra showing relative signal intensities of syringyl lignin (S_2/6_) and glucans (β-D-Glc*p*_1_) more clearly illustrates the enhanced extraction of lignin relative to alkali-insoluble cellulosic glucose in CWDHP-EGCG vs CWDHP controls (Figure 4 E-J). In addition, EGCG-derived aromatic signals (EC_6_, EC_8_, and GA_2″/6″_), which were apparent (but perhaps underestimated as noted below) before alkaline pretreatment (Figure 4C), are no longer visible after pretreatment (Figure 4D). This implies that EGCG-rich lignin fractions might be preferentially removed during alkali pretreatment of cell walls. As we previously observed [33], EGCG units were under-represented in gel NMR spectra of CWDHPs; poor NMR responses might be attributed to poor dissolution of EGCG-containing lignin (as we observed for DHPs) or dispersion of signals due to couplings at the 6,8-positions of the epigallocatechin moiety that was targeted in our ^13^C–^1^H correlation NMR experiments [32].

Overall, these results provide compelling evidence that EGCG incorporation into lignin substantially improves the delignification of cell walls during alkaline pretreatment, even under relatively mild conditions. The most plausible explanation is that EGCG enhances lignin depolymerization via cleavage of the ester linkage between its epigallocatechin and gallate moieties. Improved alkaline solubility of lignin could also be attributed to less frequent cross-linking of lignin to carbohydrate due to EGCG’s inherent ability to readily trap quinone methide intermediates to form benzodioxane structures (Figure 3). Ionization of abundant hydroxyl groups on EGCG might also contribute to lignin dissolution in alkali. Thus if successfully bioengineered into plants, a modified lignin containing EGCG could be very desirable for reducing input costs for delignifying lignocellulose during the chemical pulping of paper or the chemical pretreatment of biomass for biofuel production.

Incorporation of EGCG into lignin also enhanced the extraction of carbohydrates from the cell walls with alkali; the recovery of total sugars in alkali-insoluble residues of CWDHP-EGCG was approximately 9, 21, and 23 points lower than that of CWDHP-control after alkaline pretreatment at 70, 100, and 130°C (Table 2). Both types of CWDHPs had similar glucose recovery at 70°C, but CWDHP-EGCG had lower glucose recovery than CWDHP-control at higher pretreatment temperatures. By contrast, CWDHP-EGCG always had lower recoveries of xylose, arabinose, and galactose in alkaline insoluble residues than CWDHP controls, but differences were again more pronounced at higher pretreatment temperatures. Thus high pretreatment severity (temperature) enhanced the extraction of carbohydrates, particularly from CWDHP-EGCG where more efficient removal of lignin likely aided carbohydrate exposure to and dissolution in alkali. It is also reasonable that the incorporation of EGCG reduced the level of chemical linkages between lignin and hemicelluloses and made the hemicelluloses easier to remove. It must, however, be emphasized that the alkaline extractability of carbohydrate from primary-walled CWDHPs containing high proportions of hemicelluloses and pectin will likely be more extensive than from normal plant biomass containing secondary cell walls enriched in alkali-insoluble cellulose.

### Cell wall saccharification

**Figure 5:**
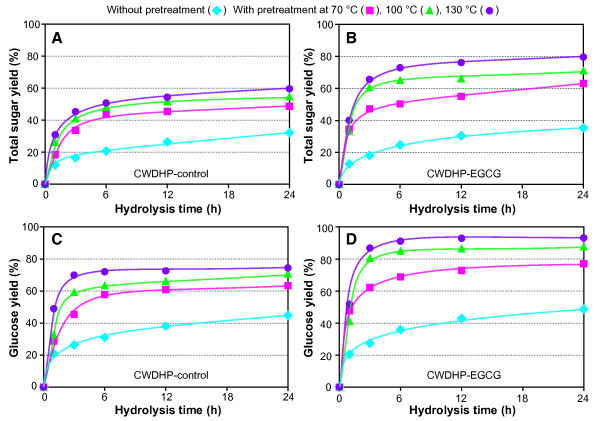
Time-course of total sugar yield (% of total sugar in the residues) and glucose yield (% glucose in the residues) during enzymatic hydrolysis of maize cell walls artificially lignified with coniferyl alcohol and sinapyl alcohol only (CWDHP-control, A and C), and in combination with epigallocatechin gallate (CWDHP-EGCG, B and D).

Finally, the enzymatic saccharification of CWDHPs, before and after alkaline pretreatment, was evaluated with cellulases supplemented with β-glucosidase and hemicellulases. The relative abundance of sugars released during saccharification was always in the order of glucose > arabinose > galactose > xylose (data not shown). Typical enzymatic hydrolysis profiles were obtained for non-pretreated and alkali-pretreated CWDHPs; saccharification was rapid during the first 3 to 6 h of hydrolysis, and continued incubation released comparatively small amounts of additional sugar (Figure 5). As illustrated in Figure 5, glucose and total sugar yields were influenced by a pretreatment X hydrolysis time interaction, with greater and more rapid sugar production from alkali-insoluble residues than from non-pretreated CWDHPs. Prior to pretreatment, average glucose and total sugar yields in the 6 to 24 h time period of maximal saccharification were similar for both types of CWDHPs (Table 3). Following pretreatment, however, glucose and total sugar yields from CWDHP-EGGC residues exceeded those from CWDHP-control residues by 10 to 21 percentage points, with the greatest differences occurring following 100 and 130°C pretreatments. Based on linear regression analysis of data in Tables 1 and 3, acetyl bromide lignin concentration on average accounted for 97% of the variation in glucose and total sugar yields from CWDHP-EGCG and their alkaline insoluble residues (*P* < 0.05); thus EGCG clearly improved saccharification by enhancing lignin removal by alkali. In contrast, sugar yields were poorly related to the lignin content of CWDHP-controls and their alkali-insoluble residues (*P* > 0.10). Removal of lignin (and possibly hemicelluloses) by alkali probably enhanced the exposure of structural polysaccharides to hydrolytic enzymes and reduced non-productive binding of these enzymes to lignin [52-56].

**Table 3:** Enzymatic and alkali-soluble (AS) glucose and total sugars released from cell wall dehydrogenation polymers (CWDHPs)

**CWDHP**	**Alkaline pretreatment (°C)**	**Enzymatic (%)**^**1**^		**Enzymatic (%)**^**2**^		**Enzymatic + AS (%)**^**2**^	
		**Glucose**	**Total sugars**	**Glucose**	**Total sugars**	**Glucose**	**Total sugars**
Control	None	37.9d	26.4d	37.9c	26.4bc	37.9e	26.4d
	70	60.7c	45.9c	46.1b	34.1ab	70.2d	59.9c
	100	66.6bc	52.3bc	44.0bc	32.2ab	77.9c	70.6b
	130	73.0b	54.9bc	40.3bc	28.3abc	85.1b	76.8b
EGCG	None	42.5d	30.2d	42.5bc	30.2abc	42.5e	30.2d
	70	72.9b	56.1b	55.6a	36.5a	79.3bc	71.6b
	100	86.3a	67.3a	44.9bc	27.4bc	92.9a	86.7a
	130	92.4a	76.2a	38.0c	22.1c	96.9a	93.1a
Analysis of variance							
CWDHP		***	*	NS	NS	***	*
Pretreatment		***	**	*	NS	***	***
CWDHP X Pretreatment Interaction		*	*	*	***	†	*

The CWDHPs were formed with monolignols (control) or with monolignols plus epigallocatechin gallate (EGCG) and pretreated with 0.075 M sodium hydroxide (15% w/w loading) at various temperatures. Data are averaged over enzymatic hydrolyses conducted for 6, 12, and 24 h.

^1^ Based on the concentration of glucose or total sugars in CWDHPs or their alkaline insoluble residues.

^2^ Based on the concentration of glucose or total sugars originally contained in CWDHPs (prior to pretreatment).

^a^ Means within columns with unlike letters differ (P < 0.05).

NS not significant; † *P* < 0.1; * *P* < 0.05; ** *P* < 0.01, *** *P* < 0.001.

When data are expressed as a proportion of sugars originally contained in CWDHPs, maximal glucose yields from alkali-insoluble residues were obtained following a mild 70°C pretreatment (Table 3). Under these conditions, glucose yields from CWDHP-EGCG residues exceeded those from CWDHP-control residues by 9.5 percentage points; both CWDHPs had a similar recovery of glucose in alkali-insoluble residues (Table 2), so yield differences were due to more extensive saccharification of CWDHP-EGCG residues (Table 3). This pretreatment also tended to maximize total sugar yields from residues, but differences between CWDHP treatments were not significant because gains in residue saccharification were offset by a lower recovery of carbohydrate in residues. More severe alkaline pretreatments at 100 and 130°C decreased glucose and total sugars yields from both CWDHPs, because gains in residue saccharification were more than offset by losses of alkali-soluble carbohydrate. If, however, alkali-soluble carbohydrate could be recovered and utilized with enzymatically released sugars, then yields of glucose and total sugars increased with pretreatment temperature and yields from CWDHP-EGCG exceeded the CWDHP-control by 9 to 16 percentage points (Table 3). Thus optimal pretreatment conditions were dependent on whether alkali-soluble carbohydrate could be utilized along with enzymatically released sugars. Optimal pretreatment conditions for a biomass crop would also likely differ from CWDHPs, but our results provide compelling evidence that EGCG incorporation into lignin substantially enhanced the ease of cell saccharification following pretreatment.

## Conclusions

In the first study, we examined the compatibility of EGCG in *in vitro* HRP-catalyzed polymerizations with CA to model how EGCG undergoes oxidative copolymerization with conventional monolignols. These studies revealed that EGCG readily copolymerized with CA to become integrally cross-coupled into the lignin polymer. Additional copolymerization studies with ethyl gallate and epigallocatechin, used to model the gallate and flavonol moieties in EGCG, suggested the former coupled with CA to mainly form benzodioxane structures, while the latter possibly participated in several coupling modes in addition to the pathway for benzodioxanes. In the second study, we artificially lignified primary maize cell walls to investigate the impact of EGCG on the lignification of cell walls and their subsequent delignification by alkali and saccharification by hydrolytic enzymes. When added with CA and SA, EGCG readily formed wall-bound lignin that was much more extensively removed by alkaline pretreatment (73 to 90%) than lignin formed only with normal monolignols (44 to 62%). Improved delignification may be attributed to cleavage of ester intra-unit linkages within EGCG, and therefore in the backbone of the modified lignin polymer, and to efficient internal trapping of quinone methide intermediates by EGCG to out-compete benzyl ether cross-linking of lignin to structural polysaccharides. Incorporation of EGCG into lignin did not influence the degradability of cell walls prior to pretreatment. Following pretreatment, alkali-insoluble residues from EGCG-lignified walls yielded up to 30% more glucose and up to 40% more total sugars than lignified controls during enzymatic saccharification. Overall, our results suggest that genetic engineering of plant metabolic pathways to permit EGCG incorporation into lignin could significantly improve the effectiveness of alkaline pretreatments and the subsequent saccharification of biomass crops.

## Methods

### General

EGCG and EGC were obtained from Biopurify (Chengdu, China) and EG was from MP Biomedicals (Solon, OH, USA). CA and SA were synthesized according to literature methods [57]. HRP (Type II, 188 purpurogallin units/mg) was from Sigma-Aldrich (St. Louis, MO, USA). Novozymes (Franklinton, NC, USA) generously provided cellulase (NS50013), β-glucosidase (NS50010), a multi-carbohydrase complex (NS50012), and xylanase (NS50030) for cell wall hydrolyses. Other chemicals were purchased from Sigma-Aldrich or Fisher Scientific (Pittsburgh, PA, USA) and were used as received.

## NMR Methods

NMR spectra were acquired on Bruker Biospin (Billerica, MA, USA) AVANCE 500 (500 MHz) or AVANCE 700 (700 MHz) spectrometers fitted with cryogenically-cooled gradient probes having inverse geometry, i.e., with the proton coils closest to the sample. Spectra were processed with Bruker’s Topspin 3.1 (Mac) software, using the central solvent peaks as internal references [δ_H_/δ_C_: acetone, 2.04/29.8; dimethylsulfoxide, 2.49/39.5 ppm]. Adiabatic 2D-HSQC (‘hsqcetgpsisp2.2’) and 2D-HSQC-TOCSY (‘hsqcetgpml’) experiments for DHP samples in the solution-state [58,59], and maize cell wall samples in a gel-state [60,61], were carried out as described previously. Processing used typical matched Gaussian apodization in F2 (LB = −0.3, GB = 0.001), and squared cosine-bell and one level of linear prediction (32 coefficients) in F1. The TOCSY mixing time was 60 ms.

### HRP-catalyzed polymerization

Monolignol solutions were prepared by dissolving CA (108 mg, 0.6 mmol) or CA (72 mg, 0.4 mmol) plus EGCG (46 mg, 0.1 mmol), EGC (61 mg, 0.2 mmol), or EG (40 mg, 0.2 mmol) in acetone-water (50 ml, 1:4, v/v). After adding HRP (2 mg), monolignols in each solution were polymerized by the dropwise addition of aqueous 0.1 M hydrogen peroxide (7.2 ml, 0.72 mmol) for 1 min followed by stirring for 9 min at room temperature. Reaction mixtures from CA or CA plus EG reactions were acidified with 0.1 M HCl (10 ml) and extracted with ethyl acetate (200 mL) to recover low molecular weight synthetic lignins (DHPs). Extracts were washed with brine (3 × 50 mL), dried over sodium sulfate, and evaporated under reduced pressure to give brownish solid residues in 42% yield from the CA oxidation and 55% yield from the CA plus EG oxidation. Oxidation products from CA plus EGCG or CA plus EGC reactions were poorly soluble in ethyl acetate, so these products were quenched with ethanol (200 ml) and evaporated to dryness. Recovered solids were stirred in acetone (100 ml), filtered, and the filtrate was evaporated to recover low molecular weight DHPs as brownish solids in 55% yield from the CA plus EGCG oxidation and 50% yield from the CA plus EGC oxidation. The DHPs were dissolved in acetone-*d*_6_ for NMR analysis (500 MHz). A high molecular weight DHP was also prepared by the “end-wise” polymerization method as described previously [59]. Polymerization conditions was as follows: CA (0.8 mmol) and EGCG (0.2 mmol) in 240 ml of acetone/sodium phosphate buffer (0.1 M, pH 6.5) (1:9, v/v) and a separate solution of hydrogen peroxide (1.44 mmol) in 240 ml of water were added by peristaltic pump over a 20 h period at 25°C to 60 ml of buffer containing HRP (5 mg), and further stirred for 4 h. The reaction mixture was then acidified (pH ~2) with 0.1 M HCl and the precipitates were collected by centrifugation, washed with 0.01 M HCl (100 ml x 3) and ultrapure water (100 ml), and lyophilized to afford DHPs (77.6% yield, w/w). The DHP was poorly soluble in general organic solvents and NMR (700 MHz) was acquired in a suspension-state (~30 mg DHP in 600 μl DMSO-*d*_6_:pyridine-*d*_5_, 4:1, v/v).

### Cell wall lignification

As described previously [32], CWDHPs were prepared by adding separate monolignol and hydrogen peroxide (1.1 eq) solutions over a 9 h period to peroxidase-containing maize cell walls (125 g fresh weight, ~3.2 g dry weight) stirred in water. A normal lignin control (CWDHP-control) was prepared by adding a two-component equimolar mixture of CA (1.3 mmol) and SA (1.3 mmol) to cell walls. Similarly, an EGCG-containing lignin (CWDHP-EGCG) was prepared by adding a three-component mixture of CA (0.75 mmol), SA (0.75 mmol) and EGCG (0.5 mmol) to cell walls. After formation, CWDHPs were washed with 9:1 (v/v) acetone: water to remove non-bound dehydrogenation products, dehydrated with acetone, and then air dried. Both treatments were replicated twice in independent experiments.

### Alkaline pretreatment

Duplicate CWDHP samples (200 mg) in 50 ml Oak Ridge thermal resistant tubes were soaked for 2 h at 25°C in 0.075 M sodium hydroxide (15% w/w loading on cell wall, 50:1 v/w liquid to cell wall ratio). Tubes were then heated at 70, 100, or 130°C for 1 h, cooled, and centrifuged (1500 × *g*; 15 min) to collect alkaline insoluble residues. Residues were thoroughly washed by repeated suspension in water followed by centrifugation (1500 × *g*; 15 min) and then freeze-dried for analysis.

### Cell wall analyses

For carbohydrate analyses, CWDHPs and alkaline insoluble residues (10 mg) were treated with 0.5 ml of 72% H_2_SO_4_ at room temperature (2°C) for 2 h, and then the mixture was diluted to 3% acid concentration followed by autoclaving (121°C, 15 psi) for 1 h. After cooling to room temperature, the hydrolysate was analyzed for neutral sugars by High-performance Ion Chromatography (HPIC) [62]. In brief, the sugars were qunatified on a Dionex (Sunnyvale, CA) ICS-3000 system equipped with an integrated amperometry detector using Dionex PA1 analytic column and PA1 guard column under the conditions of column temperature 20°C, eluent flow rate 0.7 mL/min with gradient (0 → 25 min, 100% water; 25 → 35 min, 40% water and 60% 0.1 M NaOH; 35 → 40 min, 100% water), and post-column eluent (0.5 M NaOH) flow rate 0.3 mL/min for maintaining detector cell pH > 12.5. Lignin content was determined by the acetyl bromide method [63]. Briefly, cell wall samples (~10 mg) were treated with 25% acetyl bromide/glacial acetic acid solution (v/v, 1.5 mL) at 50°C for 2 h in a sonicator. After heating, the samples were ice cooled for few minutes following centrifugation at 12,000 rpm for 5 min. About 0.5 mL of the clarified supernatant was transferred to a 10-mL glass tube with stopper that contained 2 mL of 2 M NaOH, 2.4 mL of glacial acetic acid and 350 μL of 0.5 M hydroxylamine. Finally, each sample was expanded to total 10 mL with glacial acetic acid followed by vortex for few minutes. The lignin content was determined with absorption at 280 nm using 17.7 L·cm^-1^·g^-1^ as the extinction coefficient. For gel-state NMR analyses, dried CWDHPs (100 mg) were ball milled in ZrO_2_ vessels (50 ml) containing ZrO_2_ ball bearings (10 mm × 10) using a Retsch PM100 ball mill vibrating at 600 rpm (four cycles of 5 min milling followed by 5 min of cooling). The recovered ball-milled CWDHPs were then transferred into NMR tubes, swollen in DMSO-*d*_6_:pyridine-*d*_5_ (4:1, v/v), and subjected to 2D HSQC NMR (700 MHz) experiments [60,61].

### Enzymatic hydrolysis

Prior to enzymatic hydrolysis, duplicate CWDHPs and alkali-insoluble residues were shaken in 10 ml Falcon tubes at 300-rpm for 24 h at 50°C with 50 mM sodium acetate buffer (pH 4.8, 0.2%, w/v cell wall to liquid ratio). The hydrolysis was then carried out in duplicate over a 24 h period by adding a mixture of cellulase (15 filter paper unit/g glucan), β-glucosidase (30 cellobiose unit/g glucan), multi-carbohydrase complex (15 fungal β-glucanase unit/g CWDHP), and xylanase (15 Farvet xylan unit/g CWDHP). Tetracycline (0.16%, w/v) was added to prevent microbial contamination. To create enzymatic hydrolysis time profile, sample of the hydrolysate at 1, 3, 6, 12 and 24 h was taken, respectively, and supernatant was collected after centrifugation (1500 × *g*; 5 min) and analyzed for neutral sugars by HPIC.

### Statistical analysis

Data from compositional analyses, alkali pretreatment, and enzymatic hydrolyses were subjected to an analysis of variance by the PROC mixed procedure (SAS Institute Inc., NC). Differences among treatment means were tested by the pdiff procedure at *P* = 0.05*.*

## Abbreviations

CWDHP: Cell wall-dehydrogenation polymer; CA: Coniferyl alcohol; DHP: Dehydrogenation polymer; EGC: Epigallocatechin; EGCG: Epigallocatechin gallate; EG: Ethyl gallate; HSQC: Heteronuclear single quantum coherence spectroscopy; HPIC: High-performance Ion Chromatography; HRP: Horseradish peroxidase; NMR: Nuclear magnetic resonance; QM: Quinone methide intermediates; SA: Sinapyl alcohol; TOCSY: Total coherence spectroscopy.

## Competing interests

The authors declare that they have no competing interests.

## Authors’ contributions

SE, YT, JG, XP, and JR designed research; SE, YT, and JG performed research; YT, JG, SE, XP, and JR analyzed data and wrote the paper. SE and YT contributed equally to this work and therefore should be considered as co-first-authors. All authors read and approved the final manuscript.

## Authors’ information

SE is a Postdoc with interest in the areas of chemical delignification, enzymatic saccharification, simultaneous saccharification and fermentation, and catalytic conversion of biomass for the production of biofuels. YT was a Postdoctoral Fellow supported by the Japan Society for the Promotion of Science (JSPS), and is currently an assistant scientist. He has interests in lignification reactions, including those producing novel lignins in specialized tissues and in transgenic plants, fluorescence-tagged molecules, radical coupling reactions, and bioenergy in general. JG is a Research Agronomist whose interests include the development and use of biomimetic cell wall models to investigate the formation of lignin and the effect of lignin composition, structure, and cross-linking on cell wall utilization. Other studies by JG are aimed at improving the yield and forage quality of alfalfa and enhancing the utilization of protein by ruminants through the use of protein-binding polyphenols in forages. XP is an Associate Professor of Bioenergy and Biomaterials. XP’s areas of interest include pretreatment and fractionation of lignocellulose, chemical and enzymatic saccharification of lignocellulose, biofuels (e.g. ethanol and hydrocarbon) from lignocellulose, and cellulose, hemicellulose and lignin based materials. JR is a Professor of Biochemistry and the Plants Area Leader of the DOE Great Lakes Bioenergy Research Center. His interests are in: general plant cell wall (CW) chemistry/biochemistry; lignin biosynthesis (including pathway delineation); lignin chemistry and reactions; synthesis of biosynthetic products, precursors, intermediates, molecular markers, cell wall model compounds, etc.; solution-state NMR (particularly of CW components, especially lignins); cell wall cross-linking mechanisms; methods for wall structural analysis (chemical/degradative, NMR, GC-MS, etc.).

## Supplementary Material

Additional file 1 **Figure S1.** NMR spectrum of a dehydrogenation polymer from CA and EGCG. Click here for file
