# Supplementary material for: Epigallocatechin gallate incorporation into lignin enhances the alkaline delignification and enzymatic saccharification of cell walls
Source: Biotechnol Biofuels. 2012 Aug 13;5:59. doi: 10.1186/1754-6834-5-59 (PMC3477100; doi:10.1186/1754-6834-5-59)
Supplement: Additional file 1 — Figure S1. NMR spectrum of a dehydrogenation polymer from CA and EGCG. [file 1754-6834-5-59-S1.pdf]

Additional File 1

Epigallocatechin Gallate Incorporation into Lignin Enhances the Alkaline Delignification and Enzymatic Saccharification of Cell Walls — Elumalai et al.

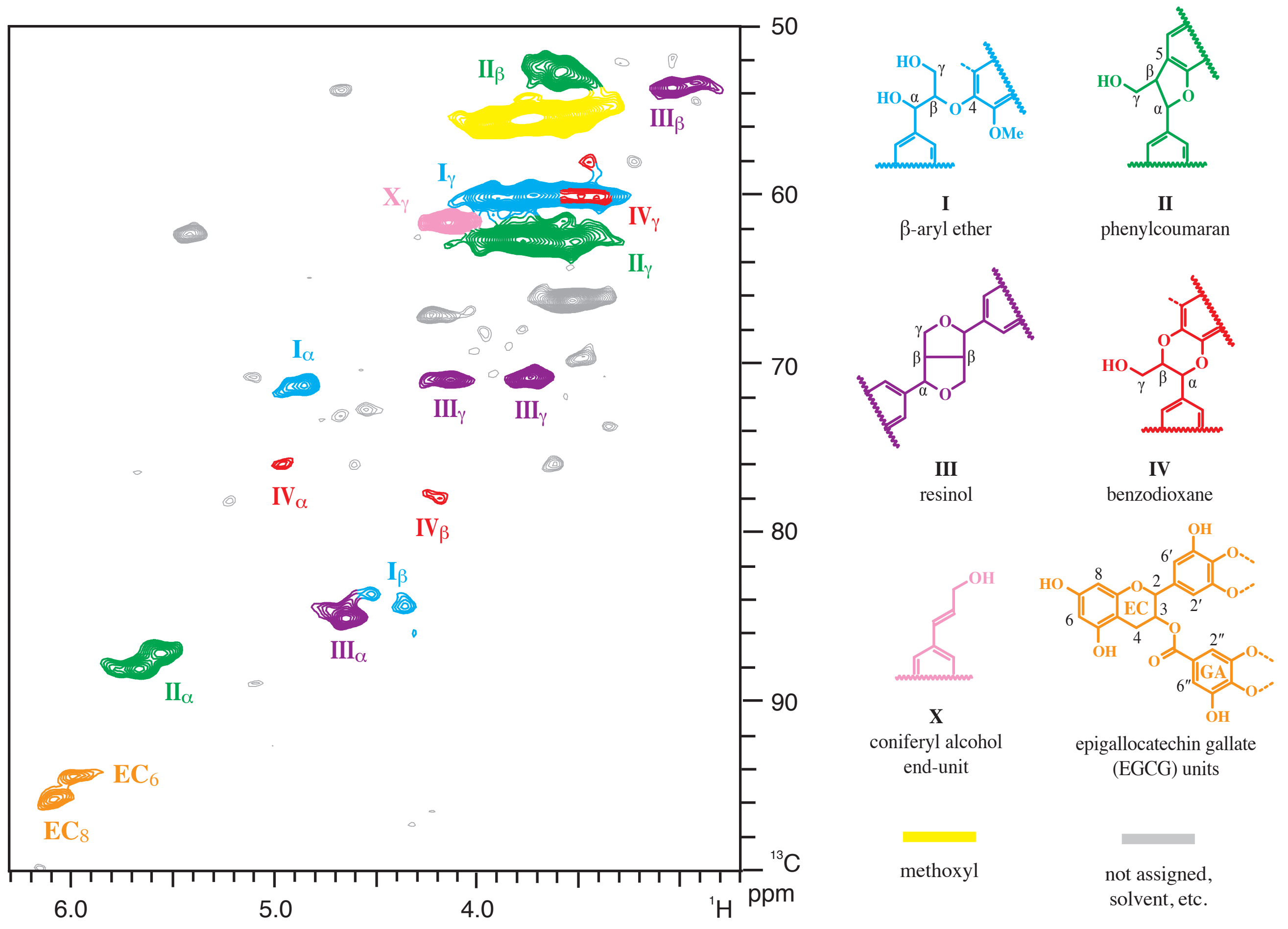

Figure S1. Partial short-range ^{13}C–^{1}H correlation (HSQC) NMR spectrum of a dehydrogenation polymer (DHP) prepared by HRP-catalyzed copolymerization of coniferyl alcohol (CA) and epigallocatechin gallate (EGCG) using the "end-wise" polymerization method.[1] The DHP was poorly soluble in general organic solvents (see main text). Therefore, the spectrum was acquired in a suspension-state (~30 mg DHP in 600 µl DMSO-d_6:pyridine-d_5, 4:1, v/v) on a Bruker AVANCE 700 Spectrometer (700 MHz).

Reference: [1] Tobimatsu Y, Davidson CL, Grabber JH, Ralph J: **Fluorescence-tagged monolignols: Synthesis and application to studying *in vitro* lignification.** *Biomacromolecules* 2011, **12:**1752-1761.
